# Flexible sensor with electrophoretic polymerized graphene oxide/PEDOT:PSS composite for voltammetric determination of dopamine concentration

**DOI:** 10.1038/s41598-021-00712-w

**Published:** 2021-10-26

**Authors:** Seung Hyeon Ko, Seung Wook Kim, Yi Jae Lee

**Affiliations:** 1 Brain Science Institute, Korea Institute of Science and Technology, Seoul, 02792 Republic of Korea; 2 Department of Chemical and Biological Engineering, Korea University, Seoul, 02841 Republic of Korea

**Keywords:** Biomedical engineering, Sensors and probes

## Abstract

We demonstrate a novel, flexible sensor with graphene oxide/PEDOT:PSS (GO/PEDOT:PSS) composite for voltammetric determination of selective low levels of dopamine. The well-distributed GO and EDOT:PSS suspension in water were deposited simply and polymerized. Consequently, the EDOT:PSS provided a strong interaction between GO and PEDOT:PSS, and it also had well-tailored interfacial properties that allowed the highly selective and sensitive determination of DA. Since the interfacial net charge is well-constructed, the sensor satisfies both the requirements of selectivity and the highly sensitive detection of low amounts of DA. In the results, the sensor with the GO/PEDOT:PSS composite exhibited a low interfacial impedance of about 281.46 ± 30.95 Ω at 100 Hz and a high charge storage capacity (53.94 ± 1.08 µC/cm^2^) for the detection of dopamine. In addition, the interference from ascorbic acid was reduced effectively to a minimum by electrostatic charge repelling of the AA and the distinct difference for the oxidation peak of the UA. Due to the fact that the GO/PEDOT:PSS composite had a net negative charge and, enhanced interfacial properties, the sensor showed a dopamine detection limit of 0.008 μM and a sensitivity of 69.3 µA/µMcm^2^.

## Introduction

Dopamine (DA) is an important biochemical molecule that is involved in learning, motor control, and motivation behavior^1^. Dysregulation of the dopaminergic and the resulting abnormal levels of dopamine are associated several neuropsychiatric diseases, including depression and schizophrenia. In addition, this operates in a delicate balance with other neurotransmitters, resulting in neurological disorders, such as Tourette’s disease^2^, schizophrenia^3^, depression^4^, and epilepsy^5^. Parkinson’s disease is one of the most well-known and important effects of DA deficiency^6^. The number of patients with Parkinson’s is in the range of 10 to 18 per 100,000 people^7^. Since DA is the major neurotransmitter used in diagnosing and treating the disease, the detection of DA, both in vivo and in vitro, has been of significant interest for clinical implications.

Currently, well-established methods for the detection of DA have been introduced, i.e., high performance liquid chromatography^8^, liquid chromatography-electrospray tandem mass spectrometry^9^, and surface enhanced Raman scattering spectroscopy and fluorescence^10^. Although they are quiet efficient, these methods have limitations, including prolonged detection time, the requirement for large volumes of samples, complex sampling, high cost, and bulky instrumentation^11^. In order to overcome these limitations, different kinds of electrochemical sensing techniques, such as differential pulse voltammetry (DPV) and chronoamperometry, have been used extensively in previous works^12,13^ due to their simple preparation, the small volume of sample that is required, and the capability for faster detection^14^.

Although the chronoamperometric sensor for the detection of DA in previous reports provided superior DA sensitivity, there was still a lack of selective detection of DA due to the close oxidation potential of other endogenous substances, such as uric acid (UA) and ascorbic acid (AA), which results in poor selectivity and poor sensitivity in the detection of DA. However, the sensor that uses the DPV technique has gained attention as a more favorable and feasible detection method because it allows the minimization of capacitive current. Thus, the Faradic current associated with the target reaction is more accurate compared to cyclic voltammetry, and a higher sensitivity can be obtained^15^. Recently, many researchers have been focused on developing modified, materials-based DPV sensor in order to obtain enhanced sensitivity and selectivity of DA detection. An interfacial electrode with negatively charged materials has been considered one of the promising strategies for achieving selective detection. On the electrode interface, which is negatively charged, the positively charged dopamine can react easily on the electrode interface, while the negatively charged interference species, such as AA are effectively repelled due to electrostatic repulsion^16^. In this respect, graphene oxide (GO) is the most attractive candidate as an electrode material for selective and sensitive detection of DA, which has been reported by many research groups due to its high surface area, electron transfer capability, biocompatibility, and biomolecular affinity. Although graphene is a 2-dimensional network sheet of sp2 hybridized carbon with unique properties^17^, there is a limitation in that chemical vapor deposition is the only way to construct devices with graphene due to its poor dispersion in aqueous and nonaqueous solvents^18^. However, the GO sheets possess relatively abundant oxygen-containing groups, such as epoxides, hydroxides, and carboxylic acid^19^. These functional groups allow the GO to disperse in aqueous solution with diverse applications, but the electrical conductivity reduced significantly due to the largely disrupted sp2 hybridized network^20^. Therefore, recently, the hybrid GO electrodes with various conductive polymers, metal nanoparticles, CNT, and others were reported to enhance the electrical conductivity of GO^21–23^. PEDOT:PSS is a conductive polymer that is used extensively, and it is a desirable candidate for making hybrid materials with GO in aqueous solution using the simple electropolymerization method. Electropolymerization can meet the requirements for an electrochemical sensor, such as simple fabrication, uniform, and mechanically more durable than previous approaches, such as spray, drop-casting, and spin coating techniques. In recent years, electropolymerization has been used extensively with carbon-based materials in liquid suspension with the aim of producing graphene-related materials^24–26^.

In this work, we demonstrate a flexible sensor with electropolymerized graphene oxide/PEDOT:PSS (GO/PEDOT:PSS) composite for the sensitive and selective voltammetric determination of low concentration of dopamine. The GO/PEDOT:PSS composite was fabricated simply as a working electrode by using the electropolymerization technique from a mixture of GO and EDOT:PSS, and it was characterized by electrochemical impedance spectroscopy (EIS), cyclic voltammetry (CV), and differential pulse voltammetry (DPV). The surface morphology, chemical state, and elemental composition of the GO/PEDOT:PSS composite were investigated using a scanning electron microscope (SEM), Fourier transform infrared spectroscopy (FT-IR), and high-resolution X-ray photoelectron spectroscopy (XPS). To our knowledge, this is the first study that simply and selectively polymerized a GO/PEDOT:PSS composite onto a thin Au electrode for the flexible DA sensor. The well-distributed GO and EDOT:PSS suspension in water were deposited simply and polymerized. Consequently, the EDOT:PSS provided a strong interaction between GO and PEDOT:PSS, and it also had well-tailored interfacial properties that allowed the highly selective and sensitive determination of DA.

Since the interfacial net charge is well-constructed, the sensor satisfies both the requirements of selectivity and the highly sensitive detection of low amounts of DA. The selective detection of DA and UA and the effective suppression of the AA response were achieved by using a DPV approach, which provides a simple way to prepare highly selective, sensitive, and flexible DA sensor applications.

## Results and discussion

### Optimization of the electropolymerization condition for the GO/PEDOT:PSS composite

**Figure 1:**
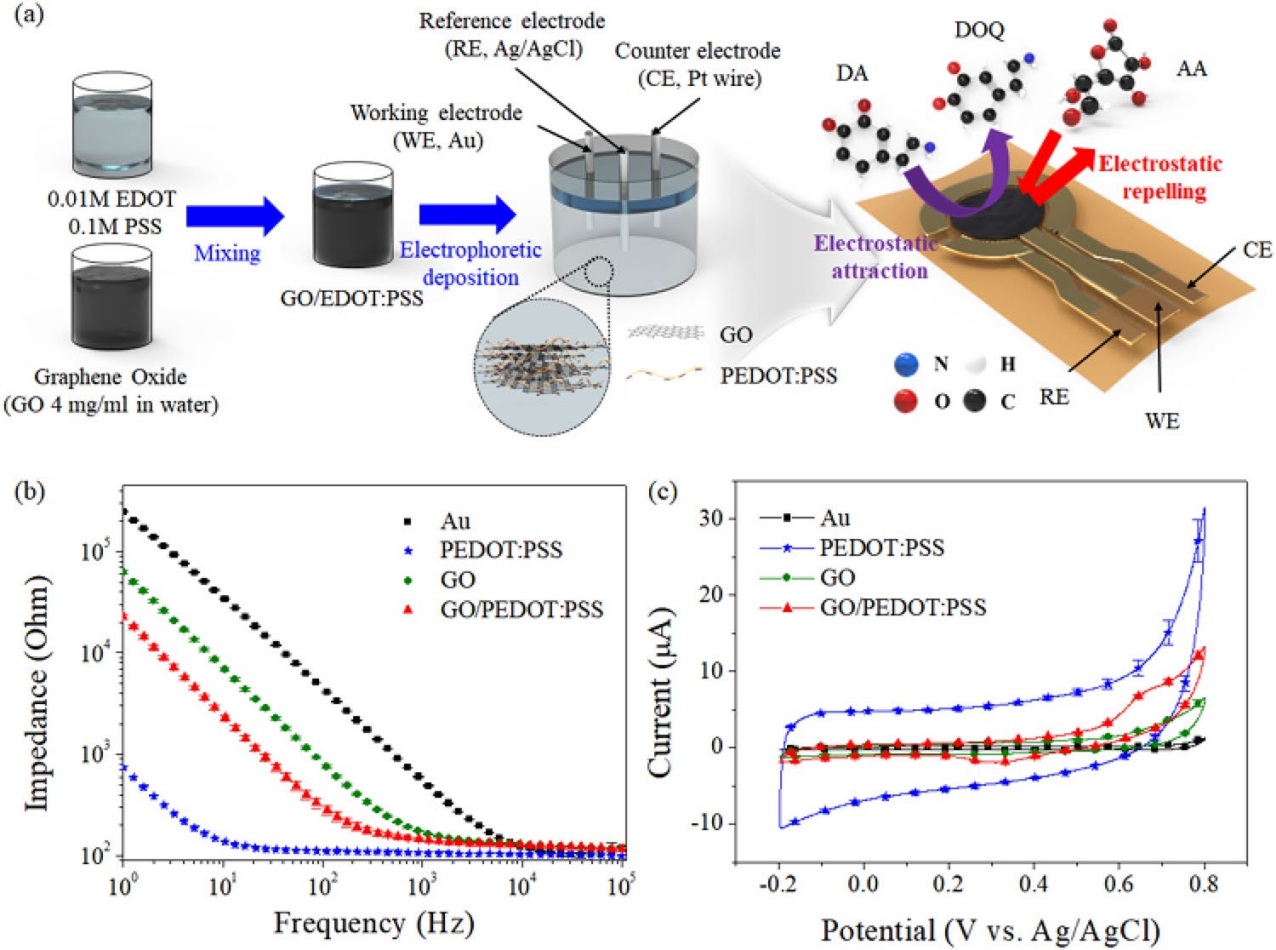
(**a**) Schematic drawing of the electropolymerization process of the GO/PEDOT:PSS composite on an Au working electrode and the configuration of the flexible sensor with the GO/PEDOT:PSS composite as a working electrode; (**b**) the comparison of the impedance and (**c**) cyclic voltammogram of Au, PEDOT:PSS, GO and GO/PEDOT:PSS composite in a 0.1 M PBS solution (pH 7.4). The scan rate was 100 mVs^−1^.

Figure 1a show the conceptual drawing for the process of fabricating the GO/PEDOT:PSS on a thin Au working electrode of the fabricated flexible sensor and the configuration of the fabricated sensor with the GO/PEDOT:PSS.

The PEDOT:PSS as a doping agent for increasing the interfacial properties of the GO layer can be obtained from electro-polymerization of the charge-balanced EDOT monomer with PSS^27^. The conductivity of PEDOT can be enhanced by doping with hydrophilic segments of PSS, which can stabilize the dispersion of EDOT:PSS in aqueous solution.

When the GO and EDOT:PSS were mixed, the PSS and EDOT chains were separated by the weakened coulombic attraction between PSS and EDOT, which was caused by addition of various functional groups (such as –COOH and –OH) of the GO domain^28^. These separated EDOT chains can interact with the GO nanosheets to extend the conductive network, which means the interaction between the GO sheets and PEDOT can help to form more conductive pathways^29^.

In order to find an optimal condition for the GO/PEDOT:PSS composite, the GO/PEDOT:PSS composites with various composite ratios (i.e., GO : EDOT:PSS = 1:1, 2:1, 5:1, and 10:1) and polymerization times (i.e., 50, 150, 300, and 600 s) were fabricated, and their impedance and CV properties were compared. Supplementary Fig. S1 shows that the interfacial impedance of the GO/PEDOT:PSS composite was increased sharply as the decrements of EDOT:PSS were added (107.62 ± 0.33, 117.89 ± 0.38, 281.46 ± 30.95, and 2429.61 ± 98.74 Ω), while the charge storage capacity (CSC) of the GO/PEDOT:PSS composite, i.e., the real activation area, was decreased as the decrements of EDOT:PSS were added (70.81 ± 0.14, 63.97 ± 0.67, 53.94 ± 1.08, and 20.36 ± 0.01 μC/cm^2^). As expected, the PEDOT:PSS, as a representative conductive polymer, affected the electrochemical properties of the GO/PEDOT:PSS composite. However, the DPV peak current responses to the various DA concentrations of the GO/EDOT:PSS composite that was prepared (i.e., 1:1, 2:1, 5:1, and 10:1) showed that the GO/EDOT:PSS compo-site with the 5:1 condition had the lowest limit of detection (0.01 μM) and linearity (R^2^ = 0.9636) in the DA concentration range up to 0.7 μM (Supplementary Fig. S2). This might be caused that the remained oxygen-containing groups of GO after polymerization (most of the negatively charged functional groups of GO are interacted with the PEDOT backbone) impart to the electrostatic interaction between composite electrode and the DA molecules^30^. In addition, the 300 s polymerization time had the lowest interfacial impedance and the highest CSC value among the other polymerization time conditions, as shown in Supplementary Fig. S3. This was caused by the increased surface activation area with time by the polymerized EDOT:PSS. In the case of a polymerization time of 600 s, the interface impedance and CSC of the GO/PEDOT:PSS composite was increased and decreased more than the 300 s condition, which might be caused by the thickly formed GO layer, which must be degraded by the interfacial properties that induce slow adsorption and electron transfer kinetics^31^. Therefore, we selected the 5:1 mixture with the 300 s electropolymerization time for the growth of the GO/PEDOT:PSS composite.

Figure 1b, c show the comparison of the impedance and cyclic voltammogram of the Au, PEDOT:PSS, GO, and the GO/PEDOT:PSS (5:1, 300 s condition) composite in a 0.1 M PBS solution (pH 7.4). The measured impedance of the Au, PEDOT:PSS, GO, and GO/PEDO:PSS composite electrodes at 100 Hz were 4123.21 ± 24.15, 111.32 ± 0.3, 750.27 ± 24.74, and 281.46 ± 30.95 Ω, respectively. In the measured CV curve, the CSC value which means accumulated charges, was expanded gradually in the order of Au < GO < GO/PEDOT:PSS < PEDOT:PSS that were 8.16 ± 0.03, 33.20 ± 0.3, 53.94 ± 1.08, and 240.98 ± 5 μC/cm^2^, respectively. Although the interfacial impedance of the GO:PEDOT/PSS composite electrode was relatively lower than the GO sheet and had a more enlarged CSC than the GO sheet due to the addition of PEDOT:PSS, their interfacial properties fell short of the lowest interfacial impedance and the largest CV curve of the PEDOT:PSS. The interfacial impedance and CSC of the GO/PEDOT:PSS composite can be controlled by adding more EDOT:PSS. The PEDOT:PSS was not appropriate for the DPV based DA determination due to brittle adhesion and low detection property (data was not shown).

**Figure 2:**
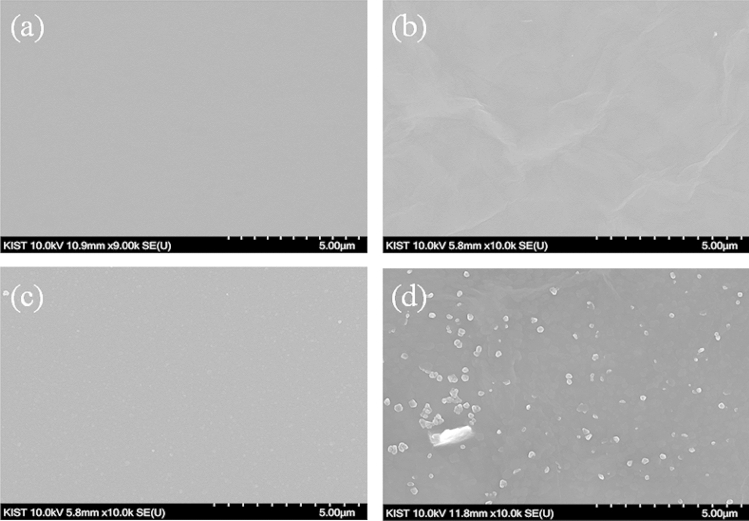
Scanning electron microscopy images of the fabricated (**a**) Au, (**b**) GO, (**c**) PEDOT:PSS, and (**d**) GO/PEDOT:PSS (mixture ratio of 5:1, polymerization time of 300 s) composite. All images display a 10,000 × magnification of the electrode surfaces. The scale bars represent 5 µm.

Figure 2 clearly shows the surface morphologies of the fabricated Au, GO, PEDOT:PSS, and GO/PEDOT:PSS (mixture ratio of 5:1, polymerization time of 300 s). The electrodeposited GO layer onto the thin Au electrode showed the typical wrinkling structure, while the pristine PEDOT:PSS layer that was polymerized onto the thin Au electrode exhibited a homogeneous distribution of the nanoparticles like grain of sand. In the surface morphology of the GO/PEDOT:PSS composite, it seemed that the PEDOT:PSS nanoparticles were well distributed along the ridges formed by the GO layer. These results must have a high correlation with the measured interfacial impedance and CV in Fig. 1b, c.

### Comparison of the structural analyses of GO, PEDOT:PSS, and the GO/PEDOT:PSS composite

**Figure 3:**
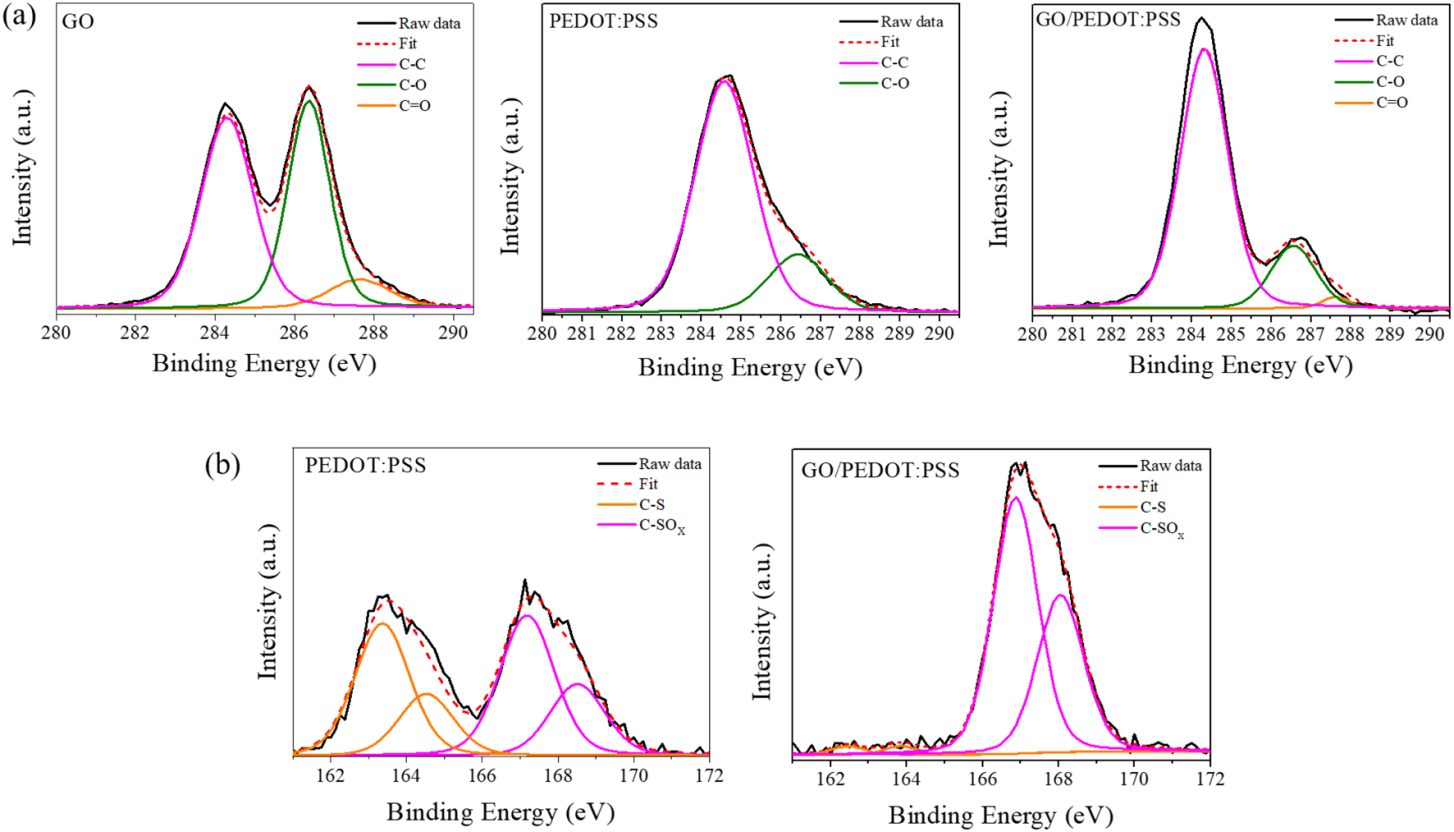
Comparison of the (**a**) C1s peaks of XPS spectra for GO, PEDOT:PSS, and GO/PEDOT:PSS composite; (**b**) Comparison of the S2p peaks for PEDOT:PSS and the GO/PEDOT:PSS composite.

The FT-IR spectra for the Au, GO, PEDOT:PSS, and GO/PEDOT:PSS were compared in Supplementary Fig. S4. The characteristic GO absorption bands appear widely at 3400 cm^−1^ (–OH), containing –OH and carboxylic acid (–COOH). The peak at 1624 cm^−1^ is referred to as the C=C stretching vibration. The peak at 1733 cm^−1^ (C=O), 1404 cm^−1^ (O–H), 1227 cm^−1^ (C–O, epoxy), and 1049 cm^−1^ (C–O, alkoxy) provide evidence of hydroxyl, carboxyl and, epoxide functional groups^32^. Conversely, the peaks in the PEDOT:PSS spectrum at 1637 cm^−1^ (C=C, alkane), 1510 cm^−1^ (C=C, aromatic stretching), and 1311 cm^−1^ (C–C) are related to thiophene rings. The peaks at 965, 826, and 682 cm^−1^ are assigned to the C–S bands of the EDOT^33^. The peaks at 1185 and 1035 cm^−1^ are known as attributed to PSS^34^. In case of GO/PEDOT:PSS transmission spectra, the absorption peak at 3400 cm^−1^ was weak compared with GO due to small amount of residual –OH in the surface of GO/PEDOT:PSS composite. The C=O and C–O stretching vibration at 1733 cm^−1^ and 1227 cm^−1^ disappeared. Also, the peak at 1624 cm^−1^ was weakened significantly, which suggested that the bulk oxygen containing functional groups of GO were reacted with EDOT and PSS. The aforementioned characteristic peak of GO, PEDOT:PSS were all reflected in spectra of the GO/PEDOT:PSS composite. The increased C–S band intensity of GO/PEDOT:PSS at 682 and 618 cm^−1^ reveals presence in the PEDOT:PSS. These results indicated that the GO/PEDOT:PSS structure was successfully constructed by GO, EDOT, and PSS nanocomposites. To clarify the notable changes of FT-IR, we carried out high-resolution XPS spectra of GO, PEDOT:PSS, and GO/PEDOT:PSS in the Fig. 3 (Fitting parameter was shown in Supplementary Table S2). Typical carbon spectra (C1s) of GO can be fitted to the three peaks at 284.6, 286.6, and 287.9 eV. These components can be assigned to the C–C, C–O, and C=O bands, respectively^35^. It is obvious that the peak intensities of C–O and C=O were strong in GO, which should be compared with the weak intensities of PEDOT:PSS. After the growth of the GO/PEDOT:PSS composite, the C–C peak had a strong intensity due to the aromatic rings of the GO/PEDOT:PSS composite. In Fig. 3b, the two sulfur S2p peaks of PEDOT:PSS at 163.8 and 165.2 eV corresponded to the –C–S–C– structure, and they were obtained in the oxidized form of thiophene (in the PEDOT chain). The peaks obtained between 168.5 and 169.7 eV represent the –C–SO_X_–C– (x: 2, 3) groups on sulfonate of PSS^36,37^. The XPS of S2p_1/2_ and S2p_3/2_ signals from the thiophene of the GO/PEDOT:PSS composite were weakened after adding GO compared to pristine PEDOT:PSS. However, the sulfur peaks of S2p1/2 and S2p3/2 from the –C–SOx–C- (x: 2, 3) groups in the GO/PEDOT:PSS composite were stronger, and they were obtained by the chemical reaction of the EDOT with –O, including functional groups of GO. The chemical composition of the composite was evaluated using atomic percent (Supplementary Table S1). The oxygen-to-carbon (O/C) ratio provides a quantitative measurement of the GO/PEDOT:PSS composite. The sulfur is present only in PEODT:PSS, and there is none in the GO sheets. With the increased O/C ratio of the GO/PEDOT:PSS composite, there was a significant increase in the content of oxygen from the large number of GO sheets rather than from the pristine PEDOT:PSS, which means that the EDOT:PSS chains were well attached by their strong interaction with GO. To explain about the state of EDOT peak with GO reaction, the O1s XPS data for PEDOT:PSS and GO/PEDOT:PSS electrode were compared in Supplementary Fig. S5. The band XPS spectra of O1s in PEDOT:PSS electrode exhibited two core-level peaks located at 529.8, 530.3 eV, which can be assigned to O=S and C–O–C for PSS and PEDOT, respectively^38^. In the GO/PEDOT:PSS, the peaks related O=S and C–O–C was similarly observed at 530.3 and 532 eV as well as the O–C=O bond by GO was observed at 534.4 eV^39^. Moreover, we can further confirm that the carboxylic acid and epoxy groups in GO act as active sites triggering the electropolymerization of EDOT. The strong binding peak for S=O/S–O peak of the GO/PEDOT:PSS might be attributed to the reaction between EDOT with GO.

### Electrochemical behavior of dopamine on the sensor with GO/PEDOT:PSS composite

In order to clear understand for the role of the electrode materials, the response currents of Au, GO, PEDOT:PSS, and GO/PEDOT:PSS in PBS containing 1 mM AA, 10 μM DA and 50 μM UA was evaluated by DPV in Supplementary Fig. S6. For the sensitive and selective DA detection, the sensor with GO/PEDOT:PSS was good enough due to selective DA detection in a low level DA concentration, while the sensor with Au, PEDOT:PSS, and GO showed some problems, such as selective DA detection issue and higher limit of detection.

**Figure 4:**
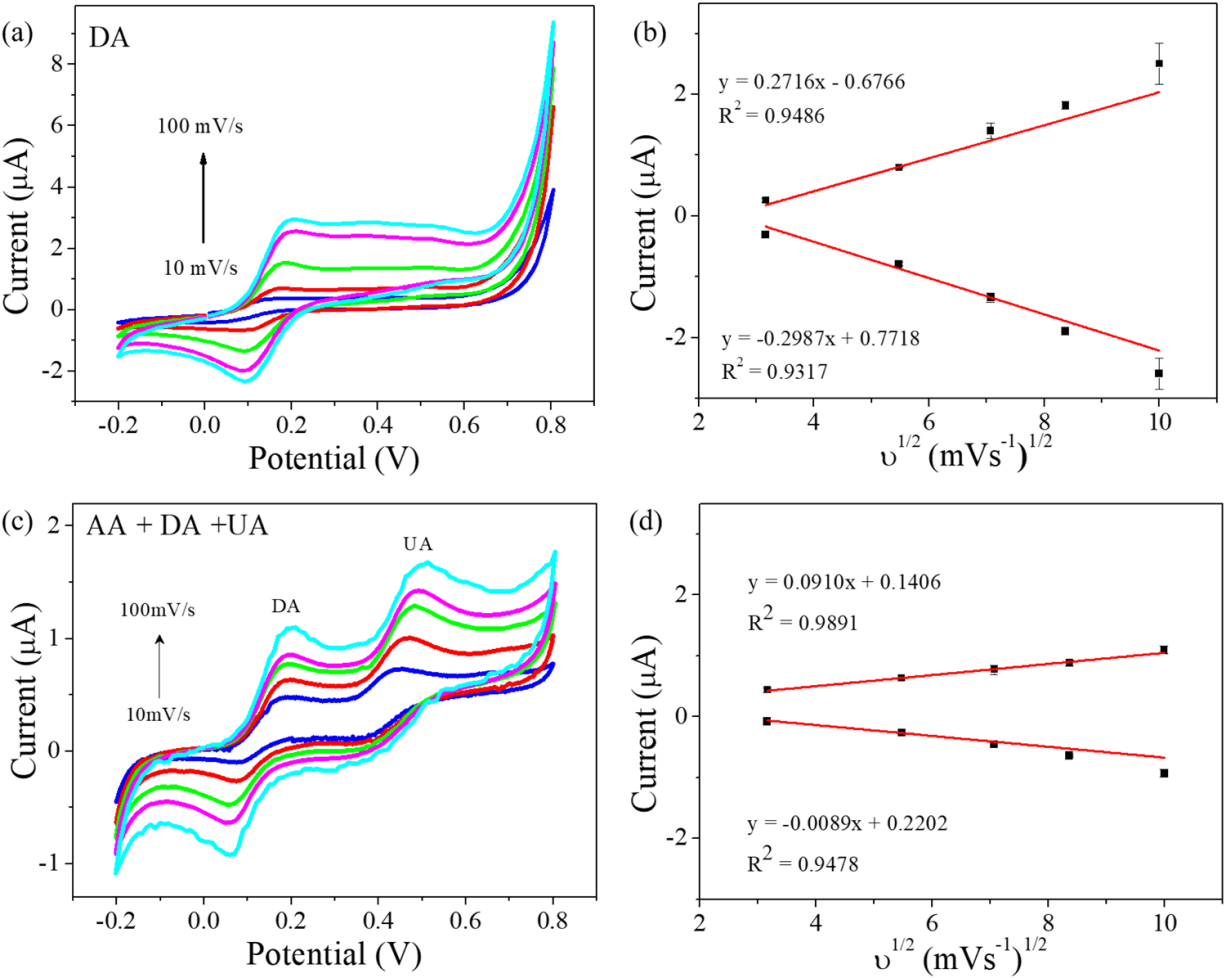
(**a**) Cyclic voltammograms of the sensor with GO/PEDOT:PSS composite to 1 mM DA concentration by various scan rates (10–100 mVs^−1^); (**b**) Linear fitting of the oxidation peak currents to the various scanning rates (n = 3); (**c**) cyclic voltammograms of the sensor with GO/PEDOT:PSS composite to the 1 mM AA, 5 μM DA, and 50 μM UA by various scan rates (10–100 mVs^−1^); (**d**) Linear fitting of the peak oxidation currents to 5 μM DA (n = 3).

The effect of pH value on the electrochemical behavior at GO/PEDOT:PSS composite was investigated in PBS (pH 5.0, 6.0, 7.4, 8.0, and 9.0) with 1 mM DA by CV as shown in Supplementary Fig. S7. The peak currents of DA was increased gradually up to pH 7.4 and the peak potential was also shifted negative potential as increment of pH value. The Supplementary Fig. S7b showed the relationship between the peak current and peak potential of DA to the different pH values. It can be found that the E_pa_ values shift negatively with increasing pH value from 5.0 to 9.0, indicating that the redox reaction of DA on GO/PEDOT:PSS were accompanied by proton transfer^40^. The linear regression equation for peak potentials and pH could be expressed as E_pa_ (V) = − 0.069 pH + 0.733 (R^2^ = 0.9990). The slope − 69 mVpH^−1^ for DA was close to a theoretical value of − 59 mVpH^−1^ that given by the Nernstian equation for equal number of two electrons and two proton transfer process^41,42^. The reaction kinetics of DA at GO/PEDOT:PSS composite to the scan rate variation was investigated by cyclic voltammetry. Figure 4a shows the cyclic voltammograms of the sensor with the GO/PEDOT:PSS composite at various scan rates (10–100 mVs^−1^) in PBS (pH = 7.4) that contained 1 mM DA. At the scan rate of 10 mVs^−1^, we measured and calculated a pair of redox peaks, the ratio of anodic and cathodic peak currents (I_pa_/I_pc_), i.e., approximately 1.05, and a peak-to-peak separation (ΔE_P_) of about 60.42 mV^41^. The anodic peak potential of DA shifted positively and the cathodic peak potential of DA shifted negatively as the scan rate increased. These results imply that the electrochemical oxidation/reduction of DA is fully reversible^44^. I_pa_ and I_pc_ as functions of scan rate were plotted in Fig. 4b. Two linear regression equations were obtained, i.e., I_pa_ (μA) = 0.2716ν^1/2^ ((mVs^−1^)^1/2^) − 0.06766 (R^2^ = 0.9486) and I_pc_ (μA) = − 0.2987ν^1/2^ ((mVs^−1^)^1/2^) − 0.7718 (R^2^ = 0.9317). To further investigate the electrochemical oxidation, the sensor with the GO/PEDOT:PSS composite was tested in the presence of 5 μM DA, 1 mM AA, and 50 μM UA at different scan rates. As shown in Fig. 4c, the oxidation current peaks of DA and UA clearly were separated based on the increment of scan rate with a peak separation (ΔE_P_) of 292 mV. The negatively charged AA (pKa = 4.10)^16^ reaction was not observed, since the surface of the GO/PEDOT:PSS has been shown to have a net negative charge due to the presence of oxygen-containing functional groups on the edges of the GO subunits^45^. It is obvious that the oxidation currents of UA and AA do not affect the determination or detection of DA. In addition, the slopes of the oxidation / reduction current peaks to the 5 μM DA were I_pa_ (μA) = 0.0910ν^1/2^ ((mVs^−1^)^1/2^) + 0.1406 (R^2^ = 0.9891) and I_pc_ (μA) = − 0.0089ν^1/2^ ((mVs^−1^)^1/2^) − 0.2202 (R^2^ = 0.9478), respectively (Fig. 4d). As the scan rate was increased, the oxidation and reduction current peaks (I_pa_ and I_pc_) increased, and anodic and cathodic peak potentials were shifted positively and negatively, respectively.

### Selective DPV detection of DA w/ and w/o interfering species on the sensor with the GO/PEDOT:PSS composite

**Figure 5:**
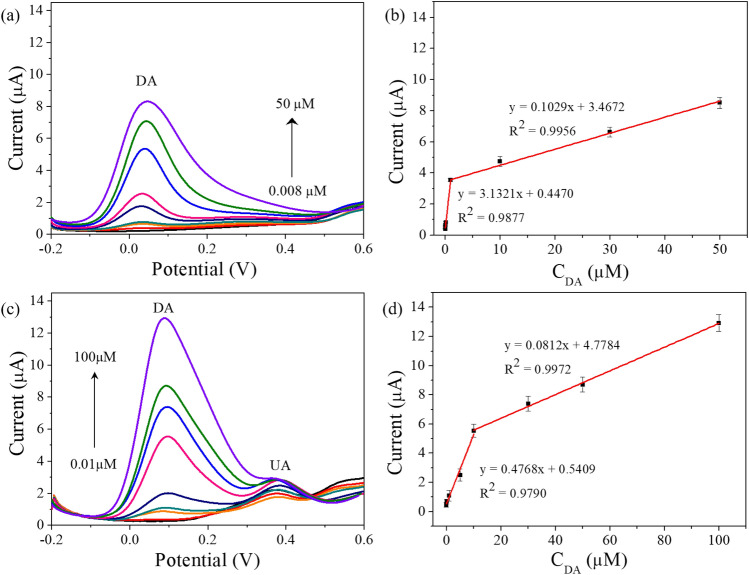
(**a**) DPV curve of the sensor with the GO/PEDOT:PSS composite to various DA concentrations (0, 0.008, 0.01, 0.1, 0.5, 1, 5, 10, 30, and 50 μM) in 0.1 M PBS (pH 7.4); (**b**) A linear fitting for the DPV oxidation peak currents to the various DA concentrations (n = 3); (**c**) DPV curve of the sensor with GO/PEDOT:PSS composite to the different DA concentrations (0, 0.01, 0.1, 1, 5, 10, 30, 50, and 100 μM) in 0.1 M PBS (pH 7.4) that contained 1 mM AA and 50 μM UA; (**d**) A linear fitting of the DPV oxidation peak currents to the various DA concentrations (n = 3).

The oxidation current responses to the various DA concentrations were investigated at the states w/ and w/o interfering species (AA and UA). Figure 5a shows the DPV responses of the sensor with the GO/PEDOT:PSS composite at different DA concentrations in PBS (pH = 7.4). The linear plot of current peak to the DA concentration showed the linear regression equation of I_pa_ (μA) = 3.1321 C_DA_ (μM) + 0.4470 (R^2^ = 0.9877), and I_pa_ (μA) = 0.1029 C_DA_ (μM) + 3.4672 (R^2^ = 0.9956) in the concentration range from 0.008 to 5 μΜ (69.3 μA/μMcm^2^) and from 5 to 50 μΜ (2.3 μA/μMcm^2^) (n = 3), respectively (shown in Fig. 5b). The limit of detection of the sensors with GO/PEDOT:PSS composite was evaluated 0.008 μΜ.

To further investigate the selective oxidation of DA without being affected by interfering species, the sensor with the GO/PEDOT:PSS composite was tested in various concentrations of DA that contained physiological concentrations of AA and UA. As illustrated in Fig. 5c, d, the oxidation current peaks showed linear responses to the change of DA concentrations (range from 0.01 to 100 μΜ) in the presence of 1 mM AA and 50 μΜ UA. The linear regression equations were expressed as I_pa_ (μA) = 0.4768C_DA_ (μΜ) + 0.5409 (R^2^ = 0.9790) and I_pa_ (μA) = 0.0812C_DA_ (μΜ) + 4.7784 (R^2^ = 0.9972) from 0.01 to 10 μΜ (10.5 μA/μMcm^2^) and from 10 to 100 μΜ (1.8 μA/μMcm^2^) (n = 3), respectively. The potential region where the DA oxidation current peak appears had shifted slightly positively, unlike when DA alone was oxidized, which might be caused by the affect of the change in the interfacial charge during the repelling of AA. An oxidation current peak by AA was not observed, and, despite the increment of the DA concentration, the oxidation current peaks by UA underwent negligible changes in a separated potential region. The performance of the fabricated flexible sensor with the GO/PEDOT:PSS composite was compared with other previous works that are shown in Supplementary Table S3. As shown in Supplementary Table S3, the sensor with GO/PEDOT:PSS composite exhibited comparable performance with reported high sensitive DA detection electrodes for the clinical level DA detection. Unlike the previous reported works, this sensor is favourable for various flexible sensor application based on high sensitive and selective DA sensor (especially free from AA oxidation) in a wide detection range.

### Reproducibility and stability of the sensor with the GO/PEDOT:PSS composite

The sensor with the GO/PEDOT:PSS composite and the GO sheet were prepared, and their responses were compared to the periodic DPV measurement, as shown in Supplementary Fig. S8. In a Supplementary Fig. S8a, it was found that the current response to 10 μM DA of the sensor with the GO sheet was decreased sharply in 10 cycles of DPV scanning (current change of 57.1%), while the sensor with the GO/PEDOT:PSS composite exhibited remarkable stability (current change of 9.02%). This might be caused by the oxygen-containing groups of the GO sheets, which allow easy swelling and disperse in water and some other solvents^46,47^. These drawbacks of the GO sheets can be overcome by combining them with PEDOT:PSS. The well-dispersed EDOT and PSS molecules in aqueous solution and the functional groups of GO may lead to strong interaction with the in situ polymerized PEDOT from EDOT. The chemical interaction between the negatively-charged, oxygen-containing groups, such as epoxides, hydroxides, and, the carboxyl groups of GO and positively charged PEDOT chains, plays a crucial role in the stability of the GO/PEDOT:PSS composites.

Supplementary Fig. S8b shows the DPV current response for sensor with PEDOT:PSS, Au, GO, and PEDOT:PSS composite at various concentrations of DA. The oxidation peak currents of DA were a linear function of its concentrations ranged from 0.01 to 0.7 μM in PBS (pH 7.4). As shown in bottom table in Fig. S8, sensor with Au and electrode exhibited low linearity and relatively higher detection limit than that of GO/PEDOT:PSS, while the sensor with GO/PEDOT:PSS showed high linearity and low detection limit. In the case of the sensor with PEDOT:PSS, although it showed high sensitivity, it’s not good enough for low level of DA.

### Real sample

In order to prove its practical feasibility, the fabricated sensor with the GO/PEDOT:PSS composite was investigated to determine the DA serum sample. All of the serum samples were diluted 50 times with PBS (pH 7.4) before measurement. No other pretreatment process was performed. Table 1 provides a comparison of the DPV peak currents to the various concentrations of DA in real samples. The recovery was in the range of 88 to 130% with a relative standard deviation (RSD, *n* = 3) of less than 6.3%. From this result, the flexible sensor with the GO/PEDOT:PSS composite exhibited sufficient performance to be used immediately for low-level DA determination applications.

**Table 1:** Detection of dopamine in serum samples.

Sample	Spiked (μM)	Found (μM)	Recovery (%)	RSD (%) (n = 3)
Serum 1	0.1	0.13	130	2.1
Serum 2	0.5	0.44	88	5.8
Serum 3	1	1.29	129	6.3

## Conclusions

The simple electropolymerized GO/PEDOT:PSS composite on a flexible sensor provides a fast and simple approach for the detection of DA by allowing the efficient fabrication of a highly sensitive and selective electrode interface. In this study, we have shown that the electropolymerization of a mixture of GO and EDOT:PSS provides a facile and effective sensor with a GO/PEDOT:PSS composite for the detection of dopamine that contains the interference species of AA and UA. The PEDOT:PSS presented a well-distributed morphology along the ridges formed by the GO layer. The PEDOT:PSS provided a strong combination for the GO layer as well as providing enhancement of the real activation area and roughness larger than the GO sheet, along with excellent electrochemical characteristics. The flexible sensor with GO/PEDOT:PSS showed a significantly improved capability for the sensitive, selective, and stable determination of dopamine (sensitivity of 69.3 µA/µMcm^2^, and a low detection limit of 0.008 μΜ), compared to the GO sheet, in terms of the DPV peak potential separation and the current peaks. The simultaneous detection of DA in the presence of AA and UA also was achieved with high selectivity, a sensitivity, and a low detection limit. In addition, through the serum sample test, the sensor with the GO/PEDOT:PSS composite proved to be relatively well matched and feasible for the practical determination of DA.

This work is the first to report a simple preparation of a flexible sensor with an electropolymerized GO/PEDOT:PSS composite, which is expected to show great promise for *in-vivo* and *in-vitro* sensing of neuro-transmitters, as well as in other integrated wearable and in vivo bioelectronics.

## Methods

### Chemicals and reagents

Polyimide (VTEC 1388) was acquired from Richard Blaine International, Inc., Philadelphia, PA, USA. DNR-L300-30 was from Dongjin, Seoul, Korea. AZ 9260 was acquired from AZ Electronic Materials, NJ, USA. Phosphate buffer saline (0.1 M PBS, pH 7.4) was obtained from Duksan general science in Korea. Graphene oxide (GO), 3,4-ethylenedioxythiophene (EDOT), poly(sodium 4-styrenesulfonate) (PSS), Dopamine hydrochloride, L-ascorbic acid (AA), and Uric acid (UA) were purchased from Sigma-Aldrich. Commercially sterile-filtered human serum (from human male AB plasma, USA origin, code H4522) was also obtained from Sigma-Aldrich.

### Fabrication of a flexible sensor with a thin Au electrode

Supplementary Fig. S9 shows the conceptual drawings for the process of fabricating the flexible sensor with Au working, counter, the reference electrode, and a photograph of the sensor that was fabricated. Briefly, the first polyimide (PI, thickness of 20 µm) as a substrate layer was spin-coated on a 4-inch SiO_2_/Si wafer. After curing in a convection oven for 10 min at 90 °C, 10 min at 110 °C, and 60 min at 210 °C, a negative photoresist (DNR-L300-30) was spin-coated on top of the PI layer for the lift-off process. After patterning using a mask aligner (MA6, Karl Suss, Garching, Germany), Cr/Au (10/100 nm) were deposited using an e-beam evaporator. After the lift-off process using acetone, the second PI was spin-coated and cured for the insulation layer (with thickness of 3 μm). The positive photoresist (AZ 9260) was coated on the second PI layer to open the electrode site and the connector pad. After patterning, the exposed PI patterns were etched by reactive ion etching (Plasma Therm, St. Petersburg, FL, USA). A laser dicing machine (M-2000, Exitech, Oxford, UK) was used to cut the perimeter of the defined sensor. Then, the flexible sensor was detached easily from the Si wafer.

### Preparation of the GO/PEDOT:PSS composite on an Au working electrode

Figure 1a shows that 0.01 M EDOT and 0.1 M PSS were mixed in deionized water. Then, the prepared EDOT:PSS solution and the GO suspension (4 mg/ml in water) were well mixed with various ratios, i.e., 1:1, 2:1, 5:1, and 10:1. The GO/EDOT:PSS mixture that was prepared was simply and selectively polymerized on the thin Au working electrode by the EPP method (4 μA current for 50, 150, 300, and 600 s). PSS typically is the dopant material that is used for PEDOT because it reinforces the structures by the bonding coulomb interaction^48^. During the polymerization from GO/EDOT:PSS to the GO/PEDOT:PSS composite, the PSS moderates the molecular entanglement into PEDOT, which might induce an improved interaction between GO and the polymerized EDOT (PEDOT). The Pt wire and Ag/AgCl electrode as counter and reference electrodes, respectively, were used for the growth of the GO/PEDOT:PSS composite. After polymerization, the composite was dried for 5 h at room temperature.

### Characterization

The electrochemical performances of the sensors were evaluated by an Autolab (PGSTAT 302 N, NOVA software, Ecochemie, Utrecht, The Netherlands) at room temperature. Three electrode configurations were used for CV, EIS, and DPV with an Au reference, Au counter, and Au (2.4 mm in diameter) or modified electrode (GO, PEDOT:PSS, GO/PEDOT:PSS) as a working electrode.

The CV with potential limits of − 0.2 and 0.8 V was performed with a scan rate of 100 mVs^−1^, and the frequency range of EIS was from 1 to 10^5^ Hz. The parameters of the DPV measurements were set as follows, i.e., the scan rate was 50 mVs^−1^, the pulse width was 0.06 s, and the amplitude was 30 mV. All solutions were prepared freshly every day and kept in the dark at 4 ℃ to avoid the oxidation of DA. All of the experiments were conducted at ambient temperature.

The surface morphologies and elemental analyses of the electrode were evaluated respectively by scanning electron microscopy (SEM, Regulus8230), Fourier transform infrared spectroscopy (FT-IR, Thermo fisher (is10) instrument), and X-ray photoelectron spectroscopy (XPS, Ulvac, Japan) with a monochromatic Al Kα X-ray source.

## Supplementary Information

Supplementary Information.
