# Supplementary material for: Flexible sensor with electrophoretic polymerized graphene oxide/PEDOT:PSS composite for voltammetric determination of dopamine concentration
Source: Sci Rep. 2021 Oct 26;11:21101. doi: 10.1038/s41598-021-00712-w (PMC8548538; doi:10.1038/s41598-021-00712-w)
Supplement: Supplementary file 1 — Supplementary Information. [file 41598_2021_712_MOESM1_ESM.docx]

**Flexible Sensor with Electrophoretic Polymerized Graphene Oxide/PEDOT:PSS Composite for Voltammetric Determination of Dopamine Concentration**

Seung Hyeon Ko^1, 2^, Seung Wook Kim^2^, and Yi Jae Lee^1*^

^1^Brain Science Institute, Korea Institute of Science and Technology, Seoul, 02792, Republic of Korea

^2^Department of Chemical and Biological Engineering, Korea University, Seoul, 02841, Republic of Korea

**(Supporting information)**

*Corresponding author email: [yijaelee@kist.re.kr](mailto:yijaelee@kist.re.kr)

**Supplementary Table. S1.** XPS surface elemental analysis parameters of GO, PEDOT:PSS, and GO/PEDOT:PSS composite.


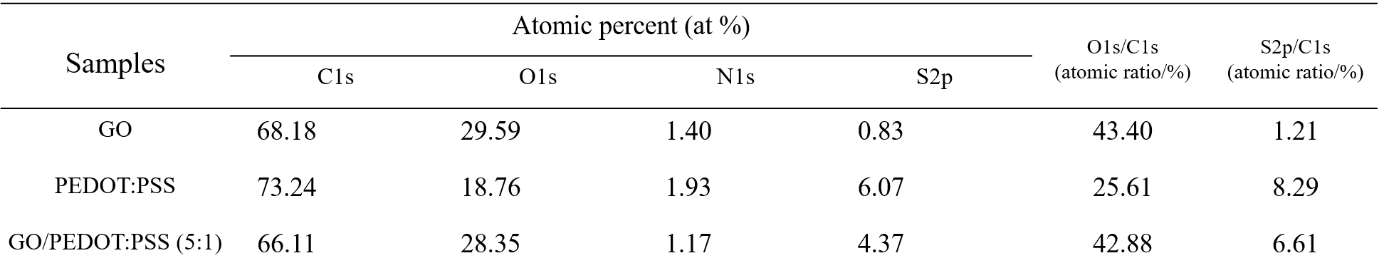


**Supplementary Table S2.** XPS fitting parameters of GO, PEDOT:PSS, and GO/PEDOT:PSS.

[GO]


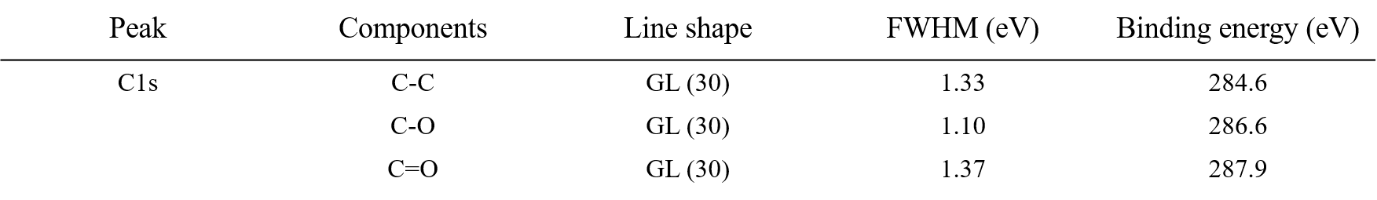


[PEDOT:PSS]


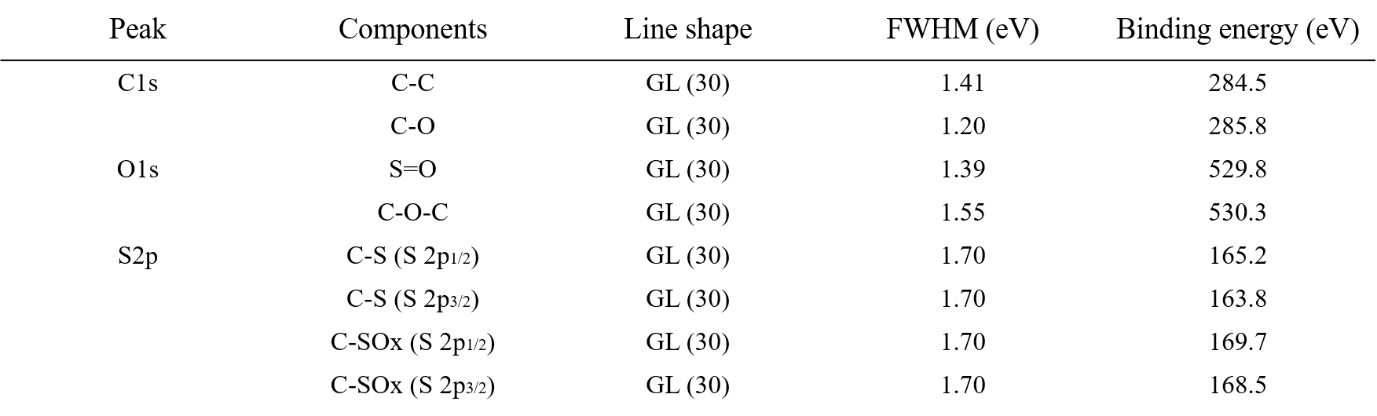


[GO/PEDOT:PSS]


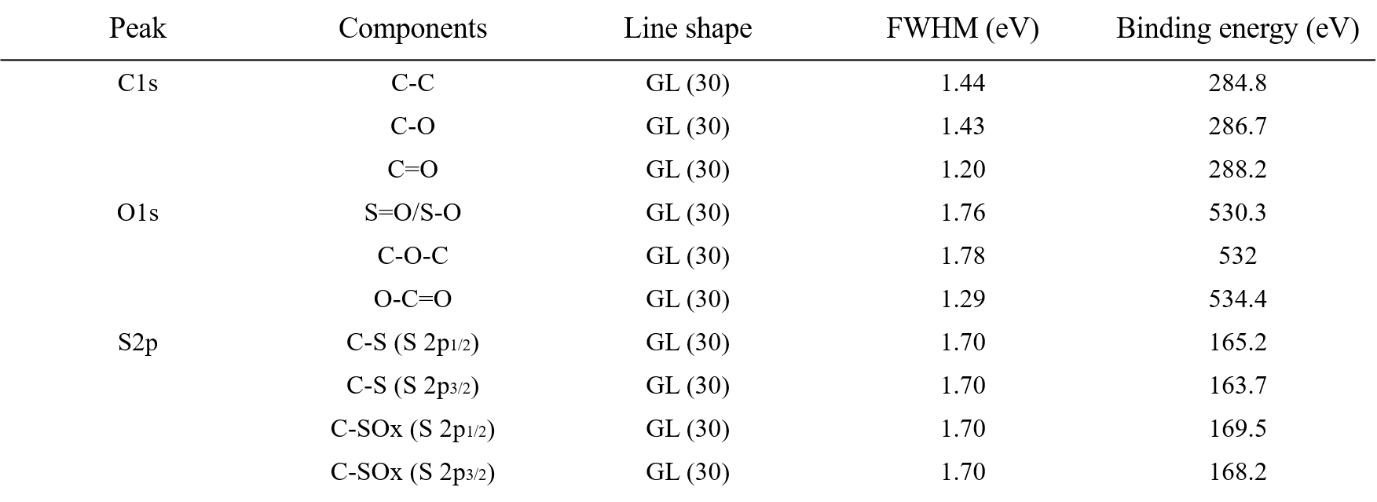


**Supplementary Table. S3.** Comparison of the specification of the DA sensor of this works and the previous works.


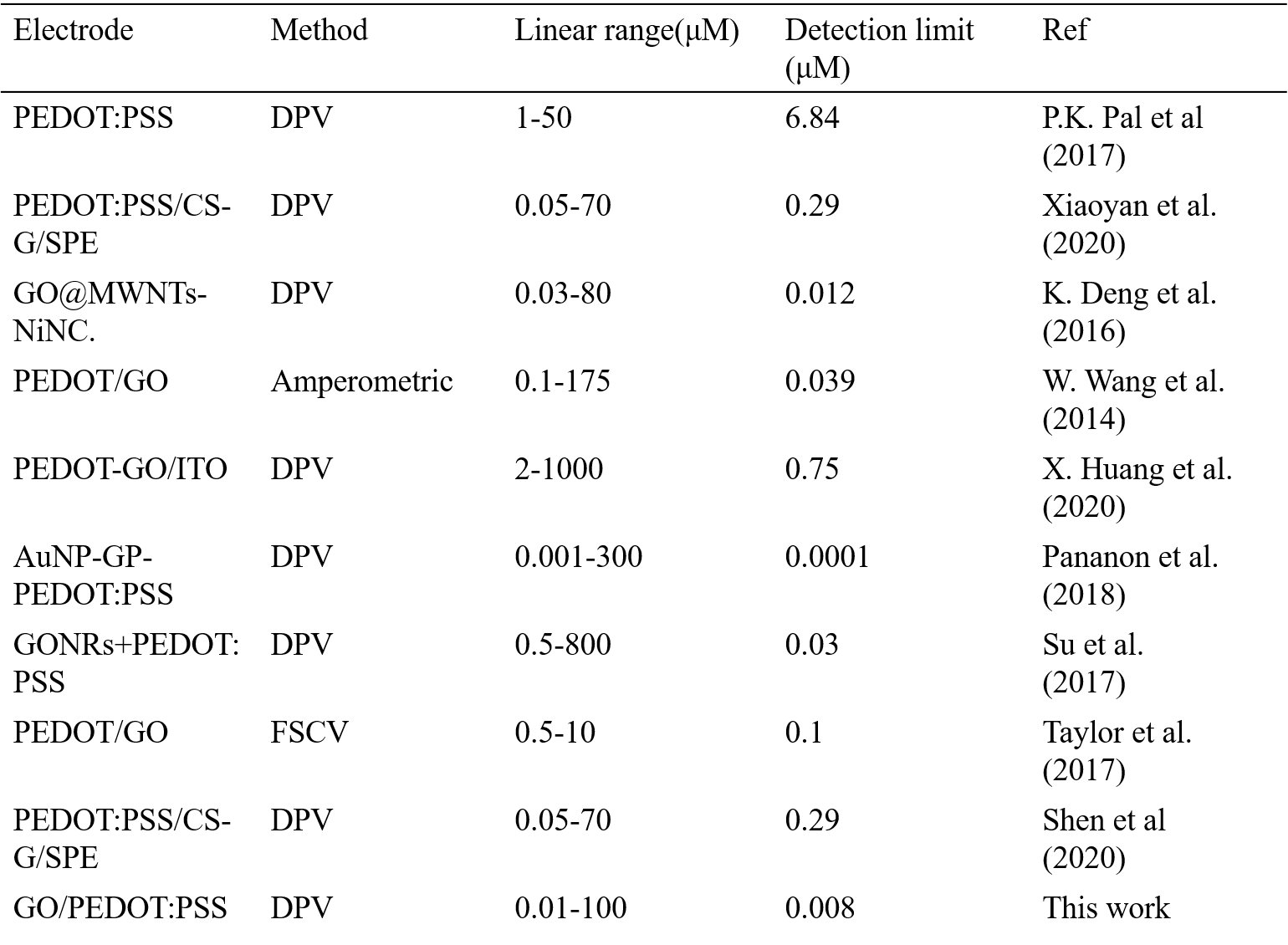


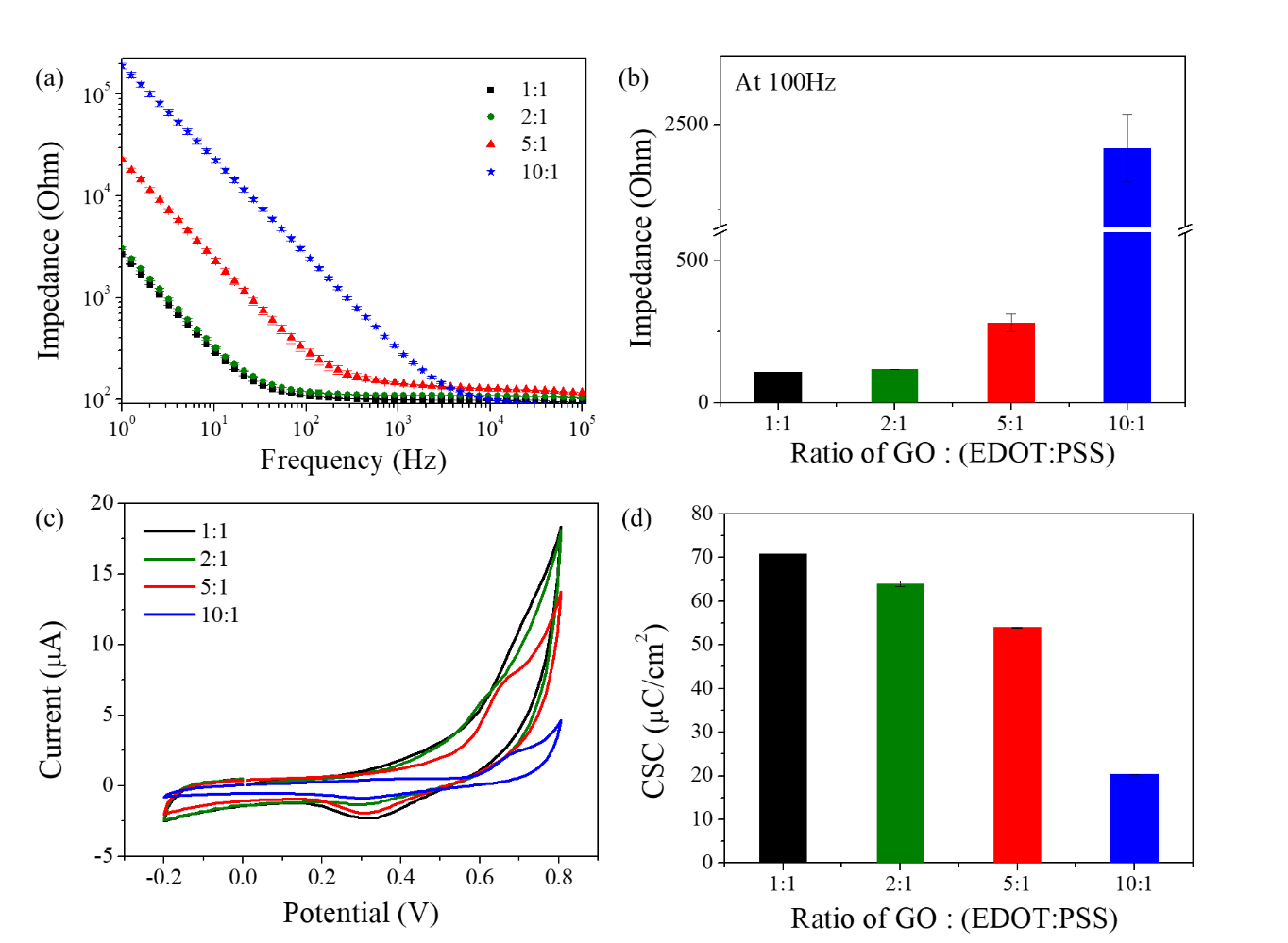


**Supplementary Fig. S1.** Comparison of (a and b) electrochemical impedance (EIS) (n=3), (c) cyclic voltammograms (CV) (n=3), and the (d) calculated charge storage capacity (CSC) of the prepared GO/PEDOT:PSS composite with mixture ratio condition of 1:1, 2:1, 5:1, and 10:1; The EIS measurement was conducted ranged from 1 to 100,000 Hz frequency in 0.1 M PBS (pH 7.4) and the scan rate of CV was 100 mV/s^-1^.


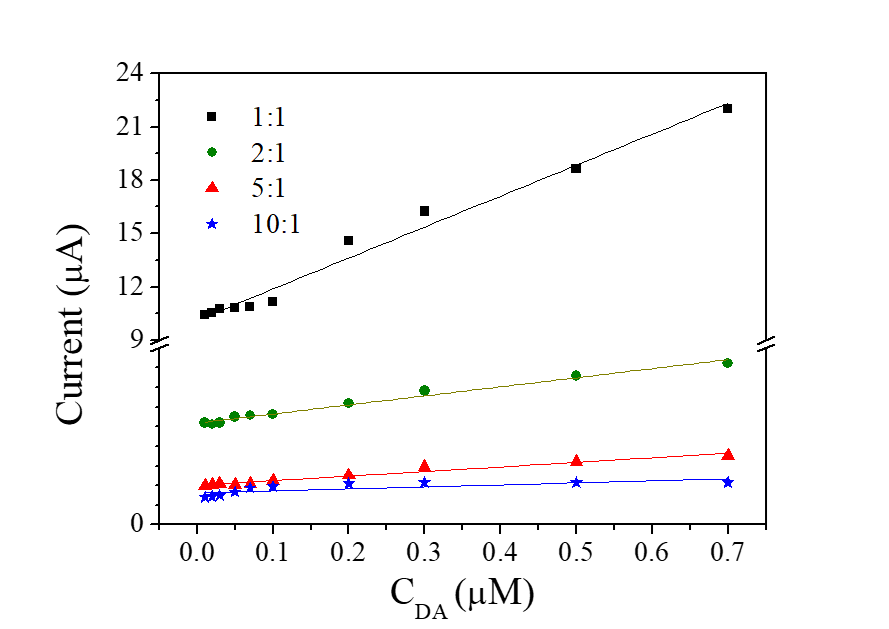


Table. Summary of the DPV response of the prepared GO/PEDOT:PSS composite with different mixture ratio to the various DA concentration.


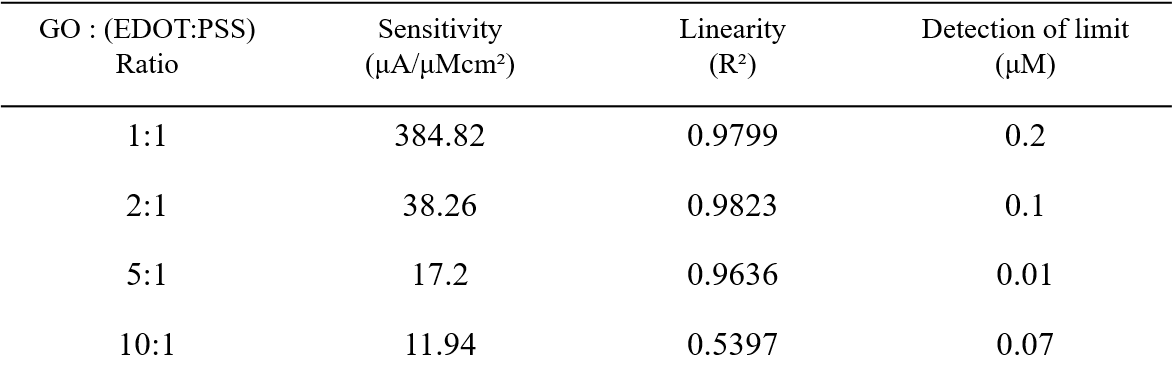


**Supplementary Fig. S2.** (Top) Comparison of the peak current responses of the prepared GO/PEDOT:PSS composite with different mixture ratio (1:1, 2:1, 5:1, and 10:1) to the DA concentration of ranged from 0.01 to 0.7 μM by differential pulse voltammetry (DPV); (Bottom table) Summary of the DPV response of the prepared GO/PEDOT:PSS composite with different mixture ratio to the various DA concentration.


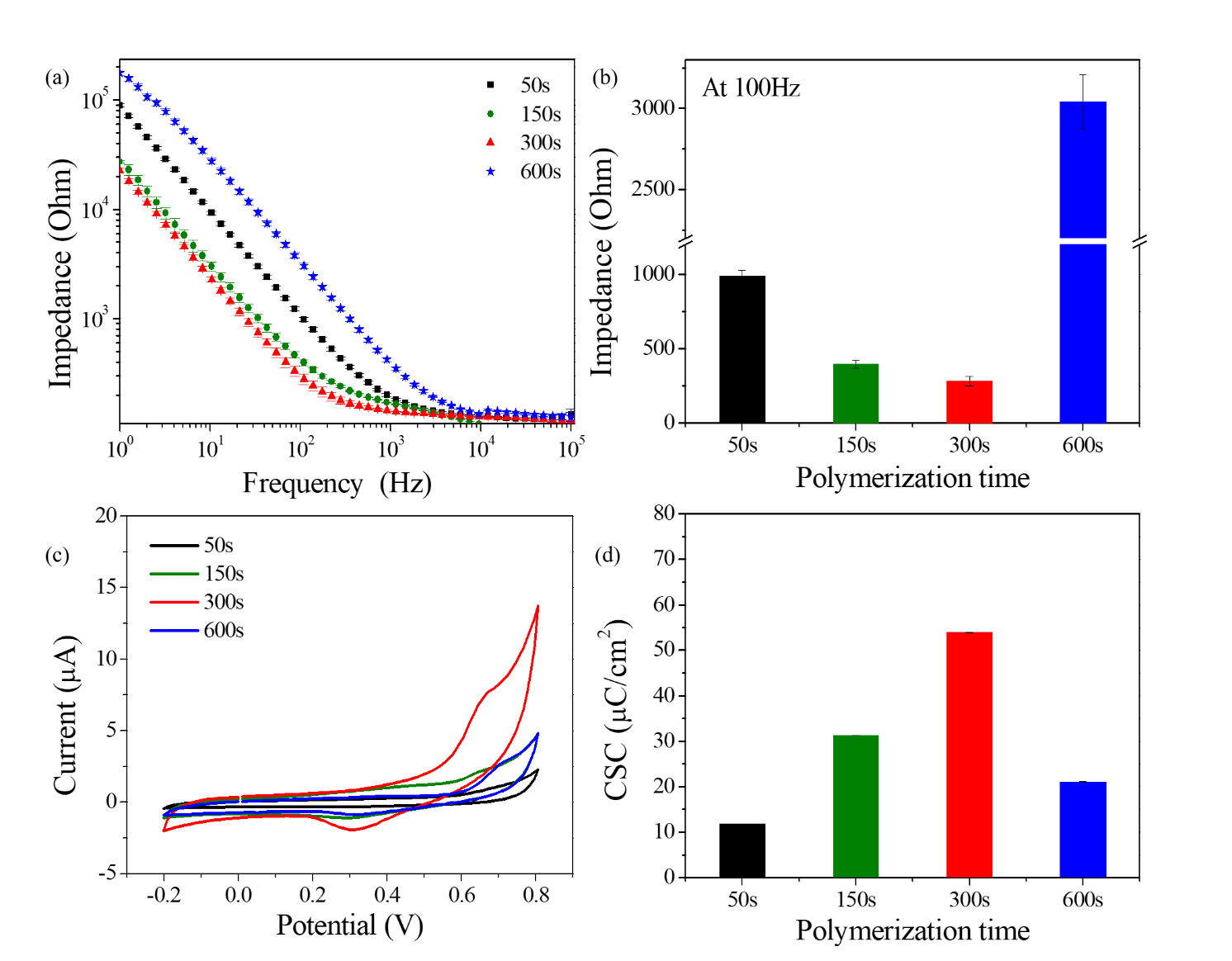


**Supplementary Fig. S3.** Comparison of (a and b) electrochemical impedance (EIS) (n=3), (c) cyclic voltammograms (CV) (n=3), and the (d) calculated charge storage capacity (CSC) of the prepared GO/PEDOT:PSS composite with polymerization time condition of 50, 150, 300, and 600 s; The EIS measurement was conducted ranged from 1 to 100,000 Hz frequency in 0.1 M PBS (pH 7.4) and the scan rate of CV was 100 mV/s^-1^.


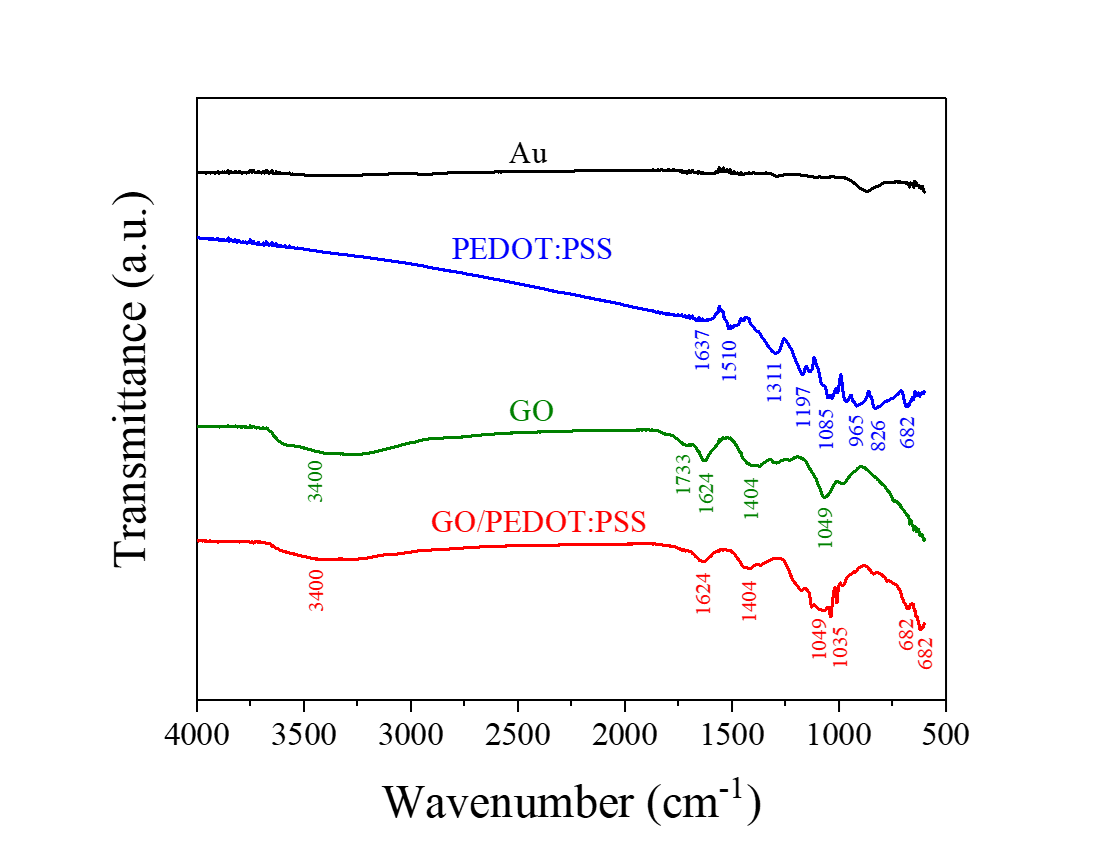


**Supplementary Fig. S4.** Comparison of FT-IR spectra of Au, PEDOT:PSS, GO, and GO/PEDOT:PSS.


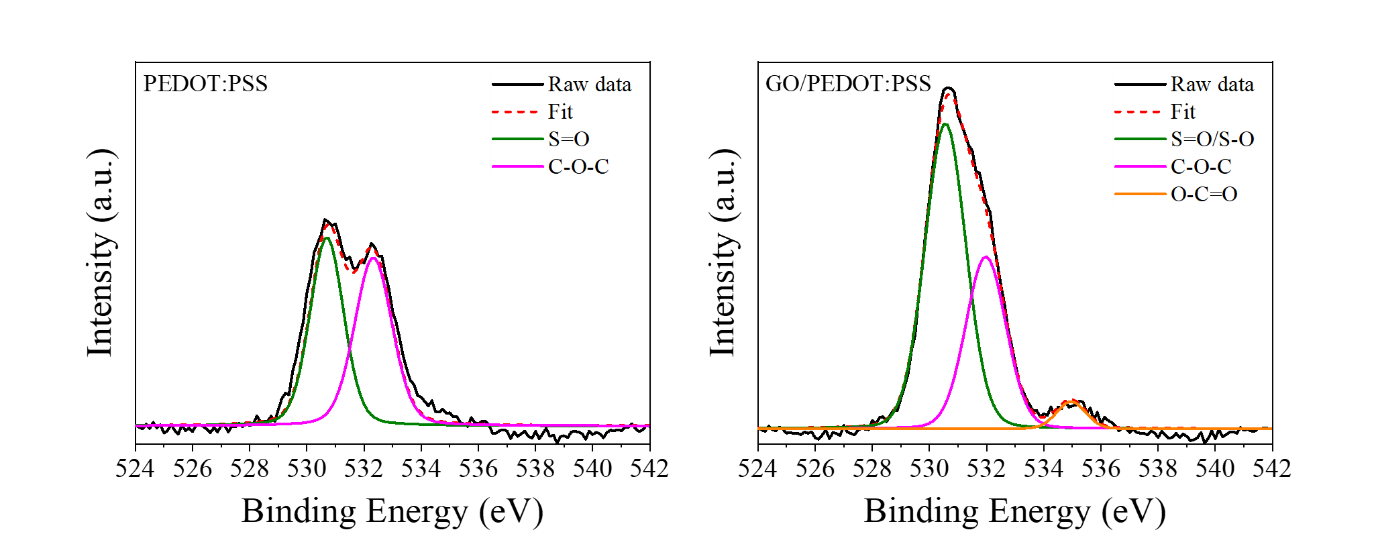


**Supplementary Fig. S5.** O1s peak of XPS spectra of PEDOT:PSS and GO/PEDOT:PSS composite deposited on gold electrode.


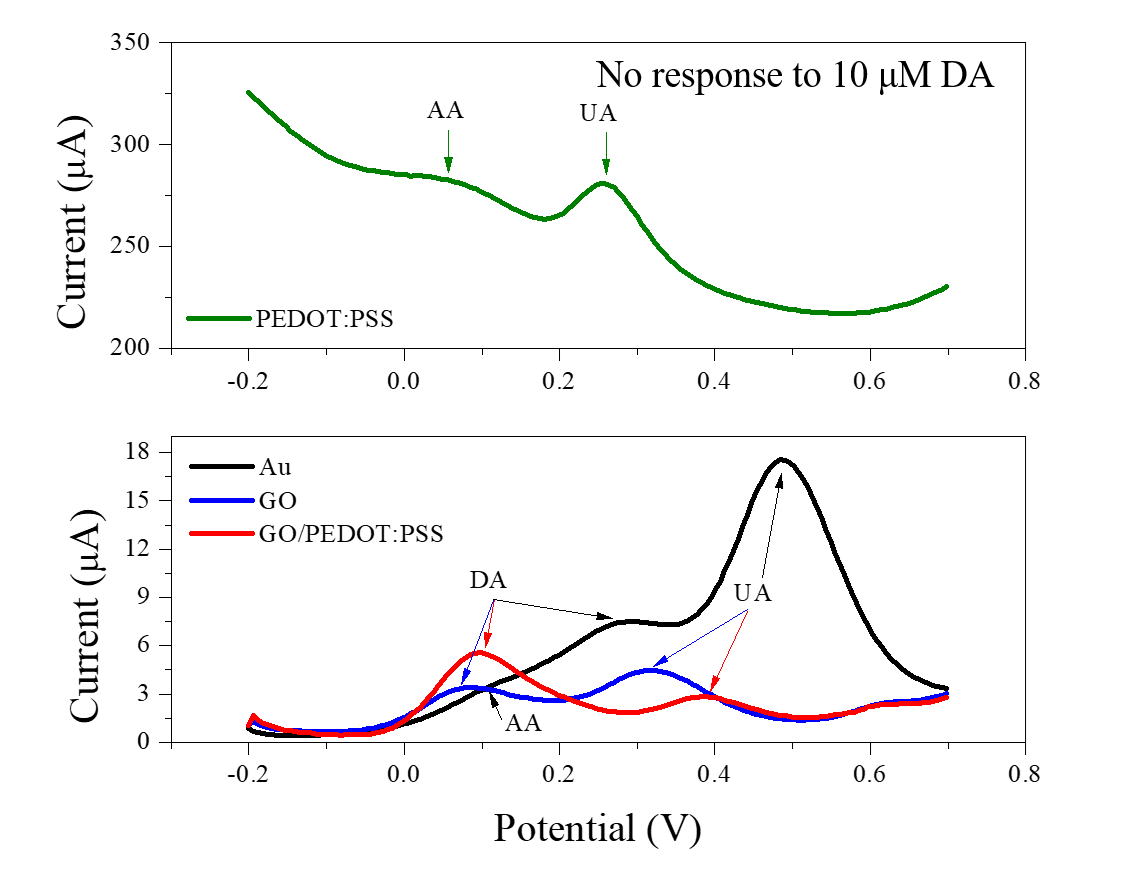


**Supplementary Fig. S6.** DPV curves of PEDOT:PSS, Au, GO, and GO/PEDOT:PSS in co-presence of 1 mM AA, 50 μM UA, 10 μM DA


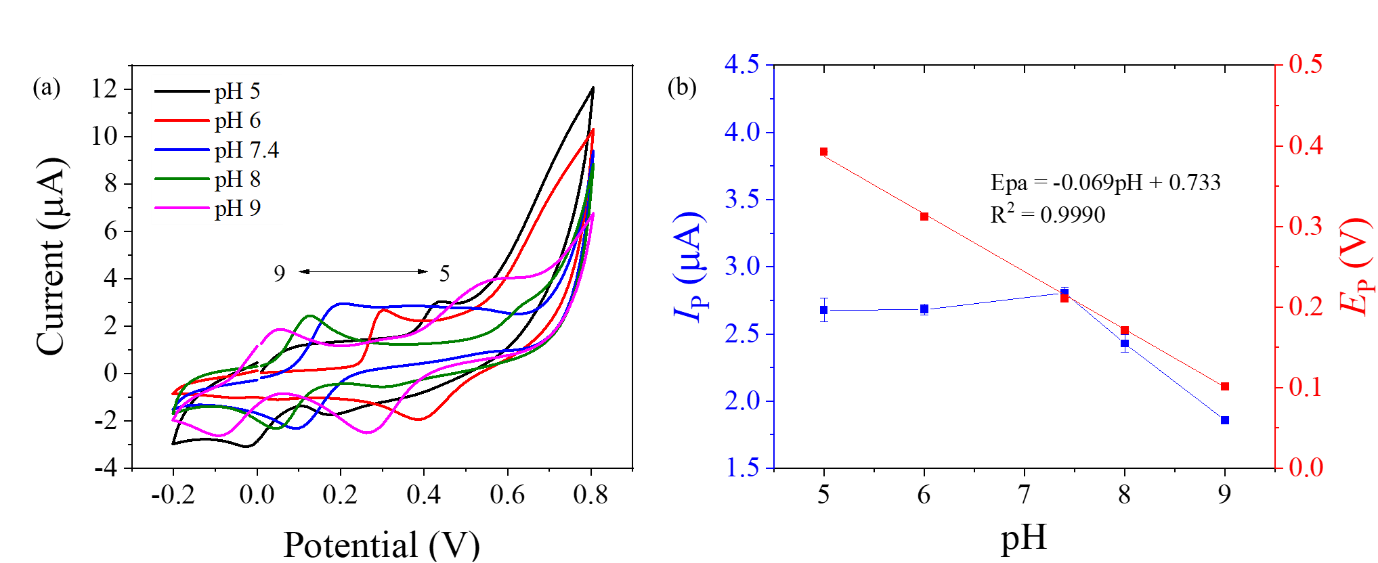


**Supplementary Fig. S7.** (a) Comparison of CV for the GO/PEDOT:PSS composite to the different pH values from 5.0 to 9.0 in 0.1 M PBS with 1 mM dopamine (scan rate of 100 mVs^-1^); (b) The relationship of (dopamine oxidation) peak current (I_p_, blue line) and peak potential (E_p_, red line) to the different pH value. Error bars represent standard deviation (n = 3).


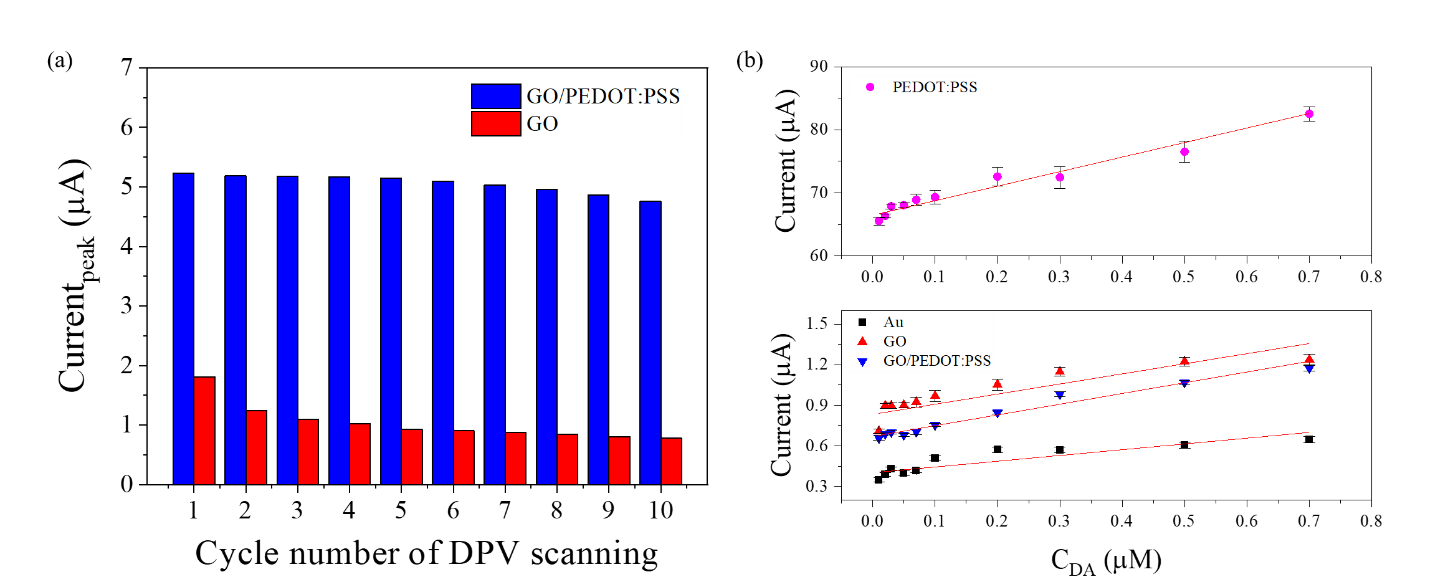


Table. Summary of the DPV response of the prepared electrodes to the various DA concentration.


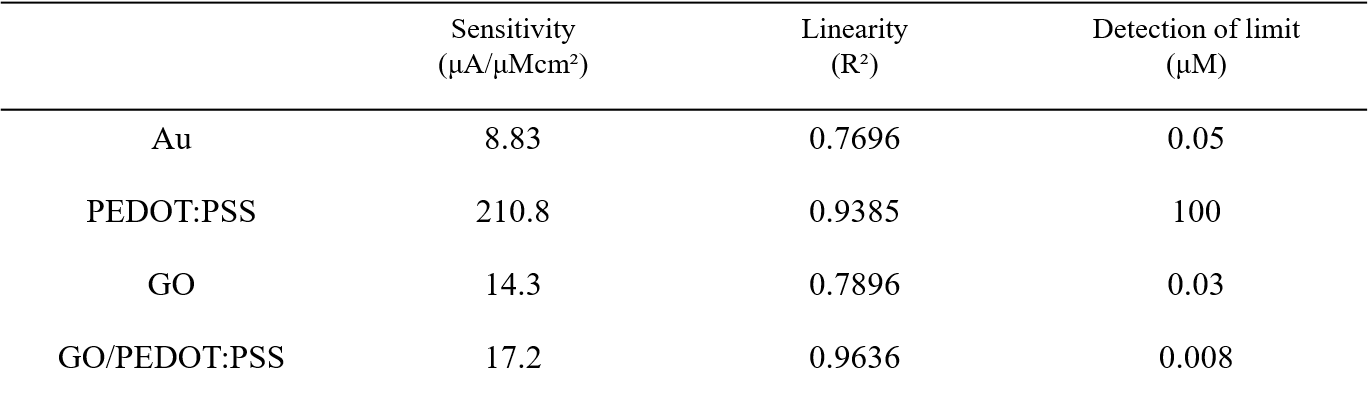


**Supplementary Fig. S8.** (a) Comparison of the oxidation peak current change at 10 μM DA concentration of GO and GO/PEDOT:PSS composite electrodes to the DPV scanning cycles; (b) Comparison of the peak current responses of the prepared GO/PEDOT:PSS composite with different electrode (PEDOT:PSS, Au, GO, and GO/PEDOT:PSS) to the DA concentration of ranged from 0.01 to 0.7 μM by differential pulse voltammetry (DPV) (n=3); (Bottom table) Summary of the DPV response of the prepared different electrodes to the various DA concentration.


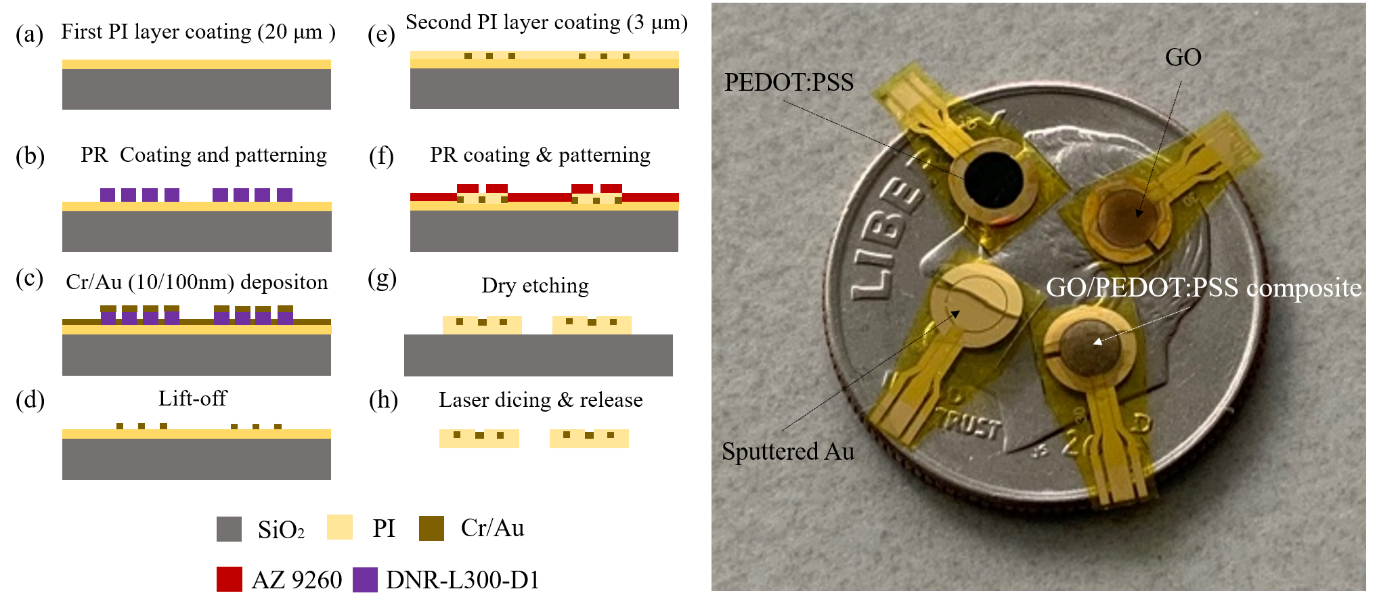


**Supplementary Fig. S9.** Fabrication sequence for the flexible DA sensor with Au working, counter, and reference electrode (Left); Fabricated flexible DA sensor with Au, PEDOT:PSS, GO, and GO/PEDOT composite electrode (Right).
